# Socioeconomic burden of pneumonia due to multidrug-resistant *Acinetobacter baumannii* and *Pseudomonas aeruginosa* in Korea

**DOI:** 10.1038/s41598-022-18189-6

**Published:** 2022-08-17

**Authors:** Chung-Jong Kim, Kyoung-Ho Song, Nam-Kyong Choi, Jeonghoon Ahn, Ji Yun Bae, Hee Jung Choi, Younghee Jung, Seung Soon Lee, Ji-Hwan Bang, Eu Suk Kim, Song Mi Moon, Je Eun Song, Yee Gyung Kwak, Shin Hye Chun, Yeon-Sook Kim, Kyung-Hwa Park, Yu Min Kang, Pyoeng Gyun Choe, Shinwon Lee, Hong Bin Kim, Korea INfectious Diseases (KIND) Study Group, Sang Won Park, Chan Mi Lee, Sook-In Jung, Seong Eun Kim, Wan Beom Park, Nam Joong Kim, Sun Hee Lee, Hyunju Lee, Jeong Su Park, Young-Jun Kim

**Affiliations:** 1 Department of Internal Medicine, Ewha Womans University Mokdong Hospital, Seoul, South Korea; 2 Department of Internal Medicine, Seoul National University Bundang Hospital, Seoul National University College of Medicine, Seoul National University Bundang Hospital, 82, Gumi-ro 173 Beon-gil, Bundang-gu, Seongnam, Gyeonggi-do 13620 South Korea; 3 Department of Internal Medicine, Seoul National University College of Medicine, Seoul, South Korea; 4 Department of Health Convergence, Ewha Womans University, 52, Ewhayeodae-gil, Seodaemun-gu, Seoul, 03760 South Korea; 5 Hallym University Sacred Heart Hospital, Anyang, South Korea; 6 Division of Infectious Diseases, Seoul Metropolitan Government-Seoul National University Boramae Medical Center, Seoul, South Korea; 7 Department of Internal Medicine, Inje University Ilsan Paik Hospital, Goyang, South Korea; 8 Division of Infectious Diseases, Chungnam National University School of Medicine, Daejeon, South Korea; 9 Department of Infectious Diseases, Chonnam National University Medical School, Gwangju, South Korea; 10 Kangwon National University Hospital, Chuncheon, South Korea; 11 Department of Internal Medicine, Seoul National University Hospital, Seoul, South Korea; 12 Department of Internal Medicine, Pusan National University School of Medicine and Medical Research Institute, Pusan National University Hospital, Pusan, South Korea; 13 Department of Paediatrics, Seoul National University Bundang Hospital, Seongnam, South Korea; 14 Department of Laboratory Medicine, Seoul National University Bundang Hospital, Seongnam, South Korea; 15 Department of Internal Medicine, Wonkwang University Hospital, Iksan, South Korea; 16 Present Address: Department of Internal Medicine, Ewha Womans University Seoul Hospital, Seoul, South Korea; 17 Present Address: Department of Internal Medicine, Hallym University Chuncheon Sacred Heart Hospital, Chuncheon, South Korea; 18 Present Address: Division of Infectious Diseases, Myongji Hospital, Goyang, South Korea

**Keywords:** Bacterial infection, Health care economics

## Abstract

We aimed to estimate the socioeconomic burden of pneumonia due to multidrug-resistant *Acinetobacter baumannii* (MRAB) and *Pseudomonas aeruginosa* (MRPA). We prospectively searched for MRAB and MRPA pneumonia cases and matched them with susceptible-organism pneumonia and non-infected patients from 10 hospitals. The matching criteria were: same principal diagnosis, same surgery or intervention during hospitalisation, age, sex, and admission date within 60 days. We calculated the economic burden by using the difference in hospital costs, the difference in caregiver costs, and the sum of productivity loss from an unexpected death. We identified 108 MRAB pneumonia [MRAB-P] and 28 MRPA pneumonia [MRPA-P] cases. The estimated number of annual MRAB-P and MRPA-P cases in South Korea were 1309–2483 and 339–644, with 485–920 and 133–253 deaths, respectively. The annual socioeconomic burden of MRAB-P and MRPA-P in South Korea was $64,549,723–122,533,585 and $15,241,883–28,994,008, respectively. The results revealed that MRAB-P and MRPA-P occurred in 1648–3127 patients, resulted in 618–1173 deaths, and caused a nationwide socioeconomic burden of $79,791,606–151,527,593. Multidrug-resistant organisms (MDRO) impose a great clinical and economic burden at a national level. Therefore, controlling the spread of MDRO will be an effective measure to reduce this burden.

## Introduction

Pneumonia is a complex disease with varied aetiology. To date, most studies on the burden of pneumonia focus on community-acquired pneumonia (CAP) or pneumonia in paediatric populations^1–8^. However, little is known about the burden of hospital-acquired pneumonia, particularly pneumonia due to multidrug-resistant organisms (MDRO)^9^.

Currently, the known causative organisms of nosocomial pneumonia are mainly *Pseudomonas aeruginosa*, *Klebsiella pneumoniae*, *Staphylococcus aureus*, and *Acinetobacter baumannii*^10–16^. Unlike in CAP, these causative agents of nosocomial pneumonia are often antibiotic-resistant bacteria. Infections caused by multidrug-resistant (MDR) *A. baumannii* (MRAB) or *P. aeruginosa* (MRPA) are more difficult to treat. In addition, antibiotics with a relatively high frequency of adverse events, such as colistin, are necessary in many of these cases. Accordingly, pneumonia caused by MDRO is likely to have a very high socioeconomic burden. Xiao et al. reported that pneumonia due to extended-spectrum beta-lactamase (ESBL) producing *K. pneumoniae* caused more economic loss than that due to ESBL-negative *K. pneumoniae*^17^. Xumei et al. reported that infection due to carbapenem-resistant bacteria, including *K. pneumoniae*, *A. baumannii*, and *P. aeruginosa*, showed a significantly higher burden than that due to carbapenem-susceptible bacteria in aspects of hospital cost, duration of hospital stay, and mortality^18^. Higher clinical and economic burdens have been associated with MDR infections, even in community-acquired infections^19^.

In a meta-analysis, the incidence of nosocomial pneumonia was 12.8–20.4%, and that of ventilator-associated pneumonia (VAP) was 31.4–36.1%^9^. The mortality rate of nosocomial pneumonia has been reported to be 21–37.4%^20^, and the occurrence of nosocomial pneumonia has been said to extend hospital stay by 18.0–30 days^9^. Furthermore, Klaus Kaier et al. reported that nosocomial *P. aeruginosa* pneumonia cases were associated with an additional medical cost of €19,000 compared to the medical cost in uninfected controls^21^.

Studies on pneumonia caused by MDRO are scarce^22–25^. Therefore, this study aimed to estimate the clinical and economic burden of pneumonia caused by MDR *A. baumannii* and *P. aeruginosa* nationwide by describing the associated clinical characteristics and additional costs.

## Results

### Clinical characteristics

During the 6-month study period, 136 cases of MDRO pneumonia were detected (108 cases of MRAB pneumonia [MRAB-P] and 28 cases of MRPA pneumonia [MRPA-P]). The mean age of the patients with MRAB-P and MRPA-P was 69.4 (± 16.8) years and 67.0 (± 17.1) years, respectively. Both types of bacteria were more common in male patients (MRAB-P: 65.7%, MRPA-P: 57.1%). MRAB-P occurred in the ICU and ward in 63% and 35%, and MRPA-P in 46.4% and 50% of the cases, respectively. Additionally, hospital-acquired infections accounted for 94% of MRAB-P and 71.4% of MRPA-P cases (Table 1).

**Table 1:** Clinical characteristics and outcomes of enrolled patients with pneumonia due to multidrug-resistant *Acinetobacter baumannii* and *Pseudomonas aeruginosa* for a 6-month period from ten hospitals in Korea.

Variables	MRAB-P n = 108	MRPA-P n = 28	p value
Age (years) [mean, (± SD)]	69.4 (± 16.8)	67.0 (± 17.1)	0.508
Sex (male)	71 (65.7%)	16 (57.1%)	0.398
**Location**			
Ward	38 (35.2%)	14 (50.0%)	
Intensive care unit	68 (63.0%)	13 (46.4%)	
Emergency room	2 (1.9%)	1 (3.6%)	
Out-patient clinic	0	0	
LOS (days) [mean, (± SD)]	73.2 (± 55.5)	92.6 (± 153.5)	0.516
Post pneumonia LOS [mean, (± SD)]	39.9 (± 45.0)	39.1 (± 37.6)	0.962
SOFA score (median, (IQR))	5 (2–8)	3 (1–7)	0.138
**Severity of infection**			
No SIRS	13 (12.0%)	5 (17.9%)	0.140
Sepsis	61 (56.5%)	18 (64.3%)	
Severe sepsis	12 (11.1%)	2 (7.1%)	
Septic shock	22 (20.4%)	3 (10.7%)	
**Mortality**			
In hospital	42 (38.9%)	10 (35.7%)	0.758
7 days	4 (3.7%)	4 (14.3%)	0.056
30 days	26 (24.1%)	9 (32.1%)	0.384
90 days	40 (37.0%)	11 (39.3%)	0.827

*MRAB-P* multidrug resistant *A. baumannii* pneumonia, *MRPA-P* multidrug resistant *P. aeruginosa* pneumonia, *LOS* length of stay, *SD* standard deviation, *IQR* interquartile range, *SOFA* sequential organ failure score.

Patients with MRAB-P had a mean length of stay (LOS) of 73.2 (± 55.5) days, with the mean LOS before and after the onset of pneumonia being 19.9 (± 22.8) days and 39.9 (± 45.0) days, respectively. The mean LOS for patients with MRPA-P was 92.6 (± 153.5) days, with the LOS before and after the onset of pneumonia being 44.4 (± 103.5) days and 39.1 (± 37.6) days, respectively.

The 30- and 90-day mortality rates were 24.1% and 37.0% for MRAB-P, and 32.1% and 39.3% for MRPA-P, respectively, with no significant difference between the two groups (p = 0.384 and 0.827, respectively). The 90-day mortality rate by age is shown in the web-only Supplementary Table S1.

### Matching with pneumonia caused by susceptible-organism infection or no infection

**Figure 1:**
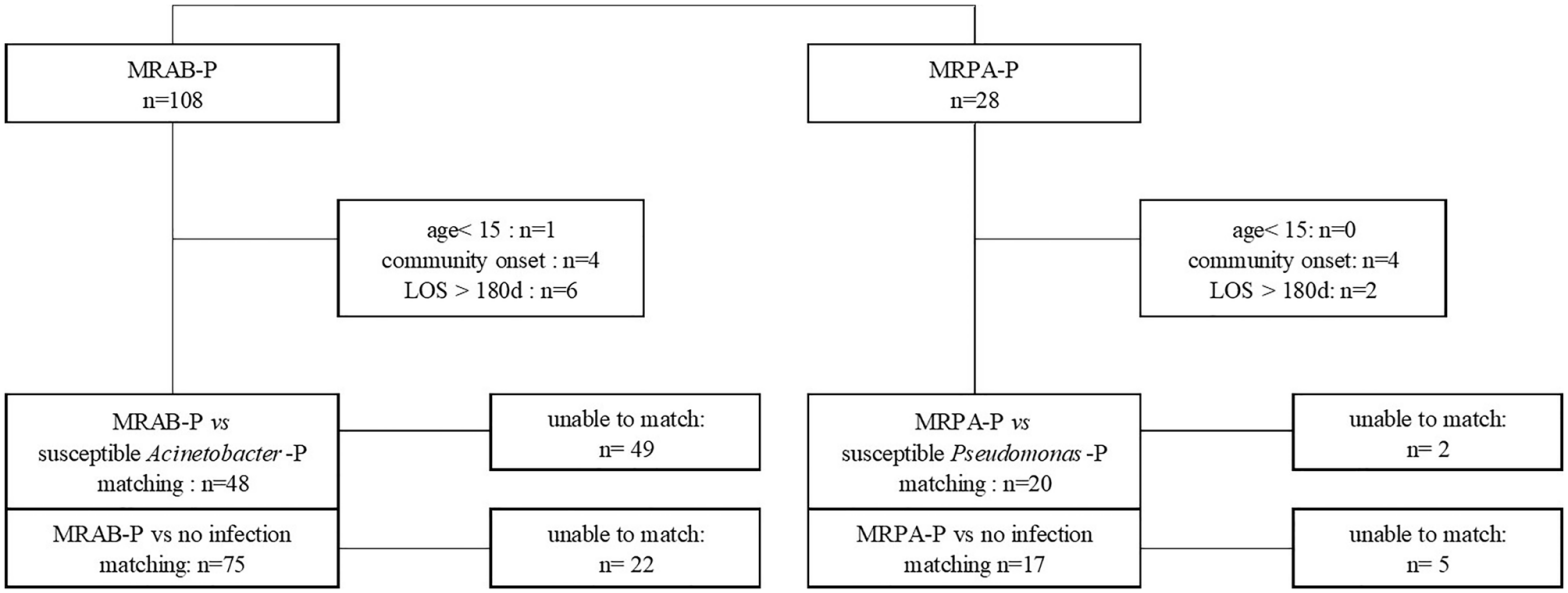
Flowchart of matching process for susceptible-organism pneumonia or no infection according to each type of pneumonia. *MRAB-P* multidrug-resistant *A. baumannii* pneumonia, *MRPA-P* multidrug-resistant *P. aeruginosa* pneumonia, *LOS* length of hospitalization, *A. baumannii*-P *A. baumannii* pneumonia, *P aeruginosa*-P *P. aeruginosa* pneumonia.

The enrolled MDRO cases (R-group) were matched with susceptible-organism infection (S-group) or no-infection (N-group) cases. A total of 54 non-MDR *A. baumannii* pneumonia (S-group) and 81 no-infection (N-group) cases were matched with MRAB-P cases (R-group). Further, 22 non-MDR *P. aeruginosa* pneumonia (S-group) and 18 no-infection (N-group) cases were matched with MRPA-P cases (R-group). Six patients with MRAB-P and two with MRPA-P in the R-group were excluded because their LOS was > 180 days. Figure 1 shows the selection flow of non-MDR bacteria pneumonia and no-infection cases in the S- and N-groups.

### Additional hospital costs and LOS: R- vs. S- or N- group

The mean difference in LOS between patients with *A. baumannii* pneumonia in the R- and S-groups was 19 (± 50.5) days, and the difference in hospital cost was $18,833 (± 33,236). The difference in the LOS between patients with *P. aeruginosa* pneumonia in the R- and S-groups was 14 (± 45.8) days, and the difference in hospital cost was $12,250 (± 33,447) (Table 2).

**Table 2:** Differences in costs and lengths of hospital stay between patients with multidrug-resistant (MDR) and non-MDR bacterial pneumonia.

Organisms	*A. baumannii*		p value	*P. aeruginosa*		p value
Group	MRAB-P (n = 48)	Non-MDR *A. baumannii* (n = 48)		MRPA-P (n = 20)	Non-MDR *P. aeruginosa* (n = 20)	
**LOS (days)**						
Mean (± SD)	60.8 (± 34.1)	41.5 (± 37.5)	0.01	55.1 (± 35.2)	40.8 (± 37.5)	0.221
Median (IQR)	53.5 (34.5–80.0)	25.7 (17.8–59.5)	< 0.01	44.0 (31.3–85.0)	28.4 (11.8–62.0)	0.126
LOS difference (days)	19.3 (± 50.5)			14.3 (± 45.8)		
**Hospital cost ($)**						
Mean (± SD)	42,484 (± 24,986)	23,651 (± 19,187)	< 0.01	40,950 (± 30,088)	28.700 (± 26,712)	0.181
Median (IQR)	41,960 (19,985–55,931)	20,000 (9174–32,627)	< 0.01	31,351 (18.486–72,316)	24,334 (4933–45,698)	0.168
Hospital cost difference ($) mean (± SD)	18,833 (± 33,236)			12,250 (± 33,447)		

*MRAB-P* multidrug resistant *A. baumannii* pneumonia, *MDR* multidrug-resistant, *MRPA-P* multidrug resistant *P. aeruginosa* pneumonia, *LOS* length of stay, *IQR* interquartile range, *SD* standard deviation.

The mean difference in the LOS between patients with *A. baumannii* pneumonia in the R-group and patients in the N-group was 48.5 (± 42.0) days, and the difference in hospital cost was $42,203 (± 35,792). The mean difference in the LOS between patients with *P. aeruginosa* pneumonia in the R-group and patients in the N-group was 44.7 (± 38.3) days, and the difference in hospital cost was $35,556 (± 31,834) (Table 3).

**Table 3:** Differences in costs and length of hospital stay between patients with multidrug resistant organisms’ pneumonia and no infection.

Organisms	*A. baumannii*		p value	*P. aeruginosa*		p value
Group	MRAB-P (n = 75)	Non-infection (n = 75)		MRPA-P (n = 17)	Non-infection (n = 17)	
**LOS (days)**						
Mean (± SD)	60.1 (± 36.0)	11.7 (± 18.6)	< 0.01	56.5 (± 37.8)	11.8 (± 6.5)	< 0.01
Median (IQR)	50.0 (33.0–80.0)	7.3 (5.5–12.3)	< 0.01	44.0 (27.5–97.0)	9.2 (6.3–15.4)	< 0.01
LOS difference (days)	48.5 (± 42.0)			44.7 (± 38.3)		
**Hospital cost ($)**						
Mean (± SD)	50,403 (± 33,090)	8200 (± 15,331)	< 0.01	43,336 (± 32,091)	7780 (± 4318)	< 0.01
Median (IQR)	47,616 (24,351–64,812)	4260 (2933–8070)	< 0.01	32,187 (14,828–77,191)	6356 (4681–11,341)	< 0.01
Hospital cost difference ($) Mean (± SD)	42,203 (± 35,792)			35,556 (± 31,834)		

*MRAB-P* multidrug resistant *A. baumannii* pneumonia, *MDR* multidrug-resistant, *MRPA-P* multidrug resistant *P. aeruginosa* pneumonia, *LOS* length of stay, *IQR* interquartile range, *SD* standard deviation.

### Caregiver cost

Patients with *A. baumannii* pneumonia in the R-group had an additional caregiver cost of $1113 for 19 days of extended hospital stay compared to the S-group. In addition, the hospital stay for the N-group was extended by 49 days, with a caregiver cost of $2869.

Furthermore, patients with *P. aeruginosa* pneumonia in the R-group had a 14-day extension of hospital stay compared to those in the S-group, with an additional caregiver cost of $820. In the N-group, the additional caregiver cost was $2635.

### Estimation of the nationwide disease burden: number of cases and productivity loss due to unexpected death

In the MDR bacteraemia study conducted together with this study, the number of patients with bacteraemia in 10 hospitals was 8.7–16.5% of the estimated number of patients nationwide (detailed estimation methods are included in the web-only supplementary results and Supplementary Table S2). Assuming that the incidence rate of MDRO pneumonia is similar, the estimated number of patients with MRAB-P in South Korea ranges from 1309 to 2483, and that of MRPA-P ranges from 339 to 644 (see web-only Supplementary Table S1). Furthermore, based on this study’s 90-day and age-specific mortality rates, the number of deaths from MRAB-P and MRPA-P was 485–920 and 133–253, respectively.

### Economic burden of pneumonia due to MDRO infection

The total additional hospital cost due to *A. baumannii* pneumonia in the R-group was $55,243,476–104,789,573 compared to the hospital cost in the N-group. When the caregiver cost and productivity loss caused by death were added, the economic burden caused by MRAB-P was $64,549,723–122,533,585.

The total additional hospital cost due to *P. aeruginosa* pneumonia in the R-group was $12,053,593–22,898,271 compared to the hospital cost in the N-group. When the cost of caregiver and social loss due to death were added, the economic loss caused by MRPA-P was $15,241,883–28,994,008 (Table 4).

**Table 4:** Results of socioeconomic burden estimation of two multidrug-resistant organisms’ pneumonia.

	MRAB-P		MRPA-P	
	Minimum	Maximum	Minimum	Maximum
Number of cases in 2017 (N)	1309	2483	339	644
Hospital cost differences (C) ($) (95% CI)	42,203 (34,102–50,303)		35,556 (20,423–50,689)	
LOS differences (L) (days) (95% CI)	49 (39–58)		45 (27–63)	
90 day mortality rate (95% CI)	37.0% (28–46%)	37.0% (28–46%)	39.3% (21–57%)	39.3% (21–57%)
Total hospital cost (NXC) ($) (95% CI)	55,243,476 (44,640,002–65,846,949)	104,789,573 (84,676,185–124,902,960)	12,053,593 (6,923,544–17,183,642)	22,898,271 (13,152,692–32,643,851)
Excess cost of caregiver use ($) (95% CI)	2869.4 (2283.8–3396.4)		2635.2 (1581.1–3689.2)	
Total cost of excess caregiver use ($) (95% CI)	3,756,005 (2,983,473–4,445,883)	7,124,644 (5,670,635–8,443,252)	893,333 (535,986–1,250,635)	1,697,069 (1,018,216–2,375,838)
Estimated number of deaths in 1 year (95% CI)	485 (365–604)	920 (693–1145)	133 (72–195)	253 (137–370)
Productivity loss due to mortality ($) (95% CI)	5,550,242 (4,178,541–6,906,609)	10,619,368 (7,994,704–13,214,252)	2,294,957 (1,240,619–3,357,129)	4,398,668 (2,374,665–6,425,870)
Total socioeconomic burden ($) (95% CI)	64,549,723 (51,802,016–77,199,441)	122,533,585 (98,341,524–146,560,464)	15,241,883 (8,700,149–21,791,406)	28,994,008 (16.545,573–41,445,559)

*MRAB-P* multidrug resistant *A. baumannii* pneumonia, *MRPA-P* multidrug resistant *P. aeruginosa* pneumonia, *CI* confidence interval, *LOS* length of stay.

## Discussion

In this study, we estimated the socioeconomic burden of pneumonia caused by MRAB and MRPA, which are common causes of nosocomial pneumonia. The additional hospital cost of MRAB-P and MRPA-P was $42,203 and $35,556, respectively. The estimated number of MRAB-P and MRPA-P cases in South Korea for 1 year was 1309–2483 and 339–644, respectively. The annual number of deaths due to MRAB-P and MRPA-P were estimated to be 485–920 and 133–253, respectively. The socioeconomic burden of MRAB-P and MRPA-P was $64,549,723–122,533,585 and $15,241,883–28,994,008, respectively.

The prognoses of MRAB-P and MRPA-P in this study were similar to those of previous studies. The proportion of MRAB and MRPA among causative agents of nosocomial pneumonia and VAP has been reported to be approximately 30–34% and 35.6%, respectively^9,26^. The previously known mortality rate of *A. baumannii* pneumonia was 37.2–48.1%^27^, and *A. baumannii* infection caused an additional economic burden of $6,693–16,074. The mortality rate of MRPA-P has also been reported to be high at 34.6%^28^, and the median ICU LOS was 34 days. In previous systematic reviews, the overall mortality rates of ICU-acquired pneumonia and VAP were 37.4% and 34.5%, respectively^9^. The LOS in ICU-acquired pneumonia was 17.7 days, and that of VAP was 30.5 days^9^. In our study, the 7-day mortality rate of MRPA-P was slightly higher than that of MRAB-P. However, there was no significant difference in long-term mortality between the two groups. A recent study showed that administering appropriate empirical antibiotics in MRAB-P contributed to reduced early mortality rates^29^. However, in our study, because the appropriateness of the initial antibiotics could not be evaluated, this could not be verified. This possibility seems to be high, given that MRAB-P usually occurs in patients who have previously had MRAB as a colonizer. Nevertheless, there was no long-term difference in mortality between the two groups because colistin, the only antibiotic currently available for MRAB-P or MRPA-P, was not as effective.

MRAB and MRPA are known to be the major causative agents of nosocomial pneumonia. However, the socioeconomic burden is also unexpected and preventable because nosocomial infections are unexpected and, in many cases, preventable. In a recent study, Andrew et al. reported that multifaceted prevention programs are cost-effective in nosocomial infections^30^. Acquiring a new disease irrelevant to the reason for hospitalisation necessitates additional medical resources and extended hospital stays, leading to socioeconomic loss. Additionally, broad-spectrum antibiotics are usually needed for MDRO infections, which can also factor in the additional disease burden. This study compared the disease burden between MDRO infection and susceptible organism infection or no infection. Even if the same bacteria caused an infectious disease, antibiotic resistance caused an additional burden. The hospital costs in cases with MRAB-P and MRPA-P (R-group) were 1.80 times and 1.42 times higher than those in cases with susceptible-organism infection (S-group), respectively, and 6.14 times and 5.57 times higher than those in cases with no-infection (N-group). Vasudevan et al. reported that the median hospital cost per day of resistant gram-negative bacterial infection in the ICU was 1.5 times higher than that of cases with no infection^31^. This is similar to our results, in which the hospital cost per day was 1.2 times higher than that in the no-infection group ($840 vs. 700 in MRAB-P and $767 vs. 659 in MRPA-P).

This study had some limitations. First, it was difficult to conclude that the difference in cost between the R- and N-groups was solely due to pneumonia because the control groups’ selection criteria were not broad. The cost difference could be ascribed only to pneumonia if the same conditions prevailed in the patient and control groups. However, in practice, it is impossible to select a control group for these patient groups in the same manner. This is a limitation of the multistate model, which has to be considered during the interpretation of the results. In addition, there is a possibility that a selection bias may affect the study results, and this remains a limitation of the study.

Second, some cases with respiratory colonization rather than pneumonia might have been included in the study because the definition of pneumonia was crude. The definition used was cases where bacteria grew in sputum, and antibiotics were used to treat them. Treatment was included in the definition because MRAB or MRPA can appear as colonized microbiota in the respiratory tract, even in the absence of pneumonia. We used this simple definition because the clinical image of infiltration on chest X-ray, fever, and abnormal blood test findings were present in many ICU patients, even if they were not diagnosed with pneumonia.

In conclusion, MRAB-P and MRPA-P infected 1648–3127 patients, resulted in 618–1173 deaths, and caused a socioeconomic burden of $79,791,606–151,527,593. MDRO impose a great clinical and economic burden at a national level. Therefore, controlling the spread of MDRO will be an effective measure to reduce this burden.

## Methods

### Study design

We prospectively collected cases of pneumonia caused by *A. baumannii* and *P. aeruginosa*. Then we matched the patients in each collected case with two control patients, one with pneumonia due to non-multidrug-resistant *A. baumannii* or *P. aeruginosa* and one with no infection. We used the multistate model utilised by Stewardson et al.^32^. The enrolled patients were categorised into three states: MDRO infection (R-group), susceptible-organism infection (S-group), and no-infection (N-group). We compared the clinical and economic aspects of the R- and S-groups, as well as the R- and N-groups. In addition, we estimated the additional burden of pneumonia caused by MDRO compared to that of susceptible-organism infection and no-infection. Our study conforms to the Consolidated Health Economic Evaluation Reporting Standards, and all procedures were carried out in accordance with relevant guidelines and regulations.

### Setting

We collected data from 10 secondary and tertiary hospitals in South Korea, which were selected based on their regional distribution. The study was performed from September 2017 to February 2018. We used a currency exchange rate of 1110 Korean won/1 US dollar for the calculations.

### Participants

We prospectively identified and collected the data of all patients with pneumonia caused by *A. baumannii*, and *P. aeruginosa*, regardless of their antibiotic susceptibilities. After collection, the R- and S-groups were selected using pre-defined criteria. In brief, multidrug resistance was defined as the non-susceptibility of *Acinetobacter* or *Pseudomonas* isolates to all three classes of antimicrobial agents, including carbapenem, aminoglycosides, and fluoroquinolones, as defined by the Korea Centers for Disease Control and Prevention. Pneumonia was defined as a disease in which bacteria grew in respiratory specimens such as sputum, transtracheal aspiration fluid, and bronchoscopy washings, and susceptible antibiotics were administered against the organisms. The selection criteria for matching S- or N-group participants were as follows: same principal diagnosis at the time of admission, same major surgery or intervention during hospitalisation, age (± 10 years), sex, and admission date within 60 days. The corresponding S- or N-group cases were selected and matched in a 1:1 ratio to the MDRO cases based on these criteria.

If a participant in the S- or N-groups experienced an invasive bacterial infection during the same hospitalisation, the participant was excluded, and another participant was selected. Additionally, any cases (R, S, or N-groups) with a total LOS of ≥ 180 days were excluded from matching.

### Variables

Data collection variables included baseline characteristics, route of admission, LOS before and after infection, and underlying disease. LOS was defined as the duration of hospitalisation from admission to discharge. Post-pneumonia LOS was defined as the duration of hospitalisation from the first day of diagnosing pneumonia to discharge. We also collected data on the severity of infection using the Sequential Organ Failure Assessment (SOFA) score and 90-day mortality. Data on hospital costs of patients in each group were also collected.

### Statistical analysis

#### Estimation of additional hospital and caregiver costs of MDRO pneumonia

We estimated the total additional direct medical cost of R-group by subtracting the mean hospital cost of the S-group or N-group from that of the corresponding R-group.

The caregiver cost was calculated by multiplying the daily fee of the hired caregiver by the extended LOS (caregiver fee was $59.1 per day [65,000 Korean won] as determined by the caregivers’ association).

#### Estimation of the number and mortality of MDRO pneumonia cases nationwide

The estimation methods are described in the online-only supplementary methods. In brief, we calculated the ratio of cases with MDRO bacteraemia between the 10 study hospitals and our previous national survey. We then assumed that the ratio of pneumonia in the 10 study hospitals to the nationwide results was the same as that of MDRO bacteraemia (unpublished data). Based on this ratio, the number of pneumonia cases and deaths nationwide was estimated.

We estimated the mortality due to MDRO pneumonia according to age distribution. First, we estimated the mortality rate for each age group among the R-group patients and then calculated the ratio of patients by age group among the total deaths. Then the estimated number of deaths in each age cohort of MDRO pneumonia on a nationwide scale was calculated by multiplying the estimated number of MDRO pneumonia cases by the 90-day mortality rate from our data.

#### Estimation of productivity loss due to death

The productivity loss due to unexpected death was calculated from the number of deaths associated with MDRO pneumonia and the annual mean wages reported by the Ministry of Labour in Korea (Labour Statistics of Korea, Ministry of Employment and Labour 2017; available from http://wage.go.kr/index.jsp). The productivity loss due to the unexpected death of a given patient was the sum of the annual wages up to the time patients would have reached 65 years of age if they had not died; the annual discount rate was 5%.

#### Estimation of the nationwide socioeconomic burden of MDRO pneumonia

The socioeconomic burden of MDRO pneumonia was estimated by summing the additional hospital cost, caregiver cost, and productivity loss due to unexpected death. The additional hospital and caregiver costs were calculated by multiplying the additional cost due to MDRO pneumonia with the estimated annual number of MDRO pneumonia patients.

### Ethical approval

This study was approved by the Institutional Review Board (IRB) of all the participating hospitals. Informed consent was waived by all IRBs, including (1) Seoul National University Bundang Hospital, (2) Ewha Womans University Mokdong Hospital, (3) Hallym University Sacred Heart Hospital, (4) Seoul Metropolitan Government-Seoul National University Boramae Medical Center, (5) Inje University Ilsan Paik Hospital, (6) Chungnam National University Hospital, (7) Chonnam National University Hospital, (8) Kangwon National University Hospital, (9) Seoul National University Hospital, and (10) Pusan National University Hospital.

### Consent to participate

Written informed consent was waived by the institutional review boards of all hospitals.

## Supplementary Information

Supplementary Information 1.

Supplementary Information 2.

## Data Availability

The datasets used and analysed during the current study are available from the corresponding author upon reasonable request.
