# Supplementary material for: Socioeconomic burden of pneumonia due to multidrug-resistant Acinetobacter baumannii and Pseudomonas aeruginosa in Korea
Source: Sci Rep. 2022 Aug 17;12:13934. doi: 10.1038/s41598-022-18189-6 (PMC9385716; doi:10.1038/s41598-022-18189-6)
Supplement: Supplementary file 1 — Supplementary Information 1. [file 41598_2022_18189_MOESM1_ESM.docx]

Supplementary Table S1. Mortality according to age group in each type of pneumonia

| Age group | Number of deaths during 6 months of study | | Estimated number of deaths in 1 year | | Estimated total number of deaths in 1 year in South Korea (maximum) | | Estimated total number of deaths in 1 year in South Korea (minimum) | |
| --- | --- | --- | --- | --- | --- | --- | --- | --- |
|  | MRAB-P | MRPA-P | MRAB-P | MRPA-P | MRAB-P | MRPA-P | MRAB-P | MRPA-P |
| 0-10 years old | 1 | 0 | 2 | 0 | 23 | 0 | 12 | 0 |
| 11-20 years old | 0 | 0 | 0 | 0 | 0 | 0 | 0 | 0 |
| 21-30 years old | 1 | 0 | 2 | 0 | 23 | 0 | 12 | 0 |
| 31-40 years old | 2 | 1 | 4 | 2 | 46 | 23 | 24 | 12 |
| 41-50 years old | 1 | 0 | 2 | 0 | 23 | 0 | 12 | 0 |
| 51-60 years old | 1 | 2 | 2 | 4 | 23 | 46 | 12 | 24 |
| 61-70 years old | 3 | 2 | 6 | 4 | 69 | 46 | 37 | 24 |
| 71-80 years old | 19 | 3 | 38 | 6 | 437 | 69 | 230 | 36 |
| 81-90 years old | 11 | 3 | 22 | 6 | 253 | 69 | 134 | 37 |
| Over 90 years old | 1 | 0 | 2 | 0 | 23 | 0 | 12 | 0 |
| Total | 40 | 11 | 80 | 22 | 920 | 253 | 485 | 133 |
| Note. MRAB-P; multidrug resistant *A. baumannii* pneumonia, MRPA-P; multidrug resistant *P. aeruginosa* pneumonia, | | | | | | | | |

Supplementary Table S2. Estimation method of nationwide numbers of multidrug-resistant *Acinetobacter baumannii* (MRAB) and *Pseudomonas aeruginosa* (MRPA) pneumonia (P)

|  | MRAB-P | MRPA-P |
| --- | --- | --- |
| Cases of MDRO pneumonia during 6-months in study hospitals (n) | 108 | 28 |
| Estimated cases of MDRO pneumonia during 12-months in study hospitals (nX2) | 216 | 56 |
| Estimated number of annual MDRO pneumonias nationwide (nX2/0.165) | 1309 | 339 |
| Estimated number of annual MDRO pneumonias nationwide (nX2/0.087) | 2483 | 644 |

Note. MDRO; multidrug resistant organism
