# Supplementary material for: Socioeconomic burden of pneumonia due to multidrug-resistant Acinetobacter baumannii and Pseudomonas aeruginosa in Korea
Source: Sci Rep. 2022 Aug 17;12:13934. doi: 10.1038/s41598-022-18189-6 (PMC9385716; doi:10.1038/s41598-022-18189-6)
Supplement: Supplementary file 2 — Supplementary Information 2. [file 41598_2022_18189_MOESM2_ESM.docx]

**Supplementary methods**

We estimated the number of pneumonia cases at the national level by the following methods. In South Korea, six kinds of multidrug-resistant bacteria have been designated as national notifiable infectious diseases and data on the number of occurrences are collected based on blood or other cultures. The corresponding strains are MRSA, VISA/VRSA, CRE, VRE, MDR Acinetobacter, and MDR *Pseudomonas aeruginosa*. For each strain, data on the number of cases isolated from blood culture and the number of cases isolated from samples other than blood are collected.

This study examined the disease burden of MDRA and MDRP pneumonia. In a concurrent study, the disease burden of MRSA, CRE, VRE, MDRA, and MDRP bacteraemia were studied together. Therefore, we estimated the occurrence proportion in the 10 hospitals included in this study, of the nationwide occurrences, based on the number of bacteraemia cases collected in the concurrent study and the number of blood cultures collected nationwide.

The number of cases of pneumonia caused by multidrug-resistant bacteria was then estimated based on the lowest and highest values ​​among these ratios. In addition, we assumed that deaths from pneumonia occurring nationwide would also occur at the same rate.

**Supplementary Results**

The number of multidrug-resistant bacteraemia cases collected over 6-months from the 10 hospitals included in this study were as follows: MRSA 260, MRAB 87, MRPA 18, CRE 20, and VRE 101. The number of cases of each type of bacteraemia in the country in 2017 were 4070, 1396, 218, 461, and 1834, respectively. Therefore, the ratio between the national level incidence and that of the 10 hospitals, based on our data, was 8.7%–16.5%.

| Variables | MRSA | MRAB | MRPA | CRE | VRE |
| --- | --- | --- | --- | --- | --- |
| Total number in sentinel surveillance in 2017 | 4,070 | 1,396 | 218 | 461 | 1,834 |
| Cases of MDRO bacteraemia during 6 months of study | 260 | 87 | 18 | 20 | 101 |
| Estimation of 1 year number of MDRO bacteraemia | 520 | 174 | 36 | 40 | 202 |
| Proportion of number of MDRO bacteraemia in the 10-hospitals: national surveillance | 12.8% | 12.5% | 16.5% | 8.7% | 11.0% |
